# Merkel Cell Carcinoma: An Update and Immunotherapy

**DOI:** 10.3389/fonc.2018.00048

**Published:** 2018-03-06

**Authors:** Hiroshi Uchi

**Affiliations:** ^1^Department of Dermatology, Graduate School of Medical Sciences, Kyushu University, Fukuoka, Japan

**Keywords:** PD-1, PD-L1, Merkel cell carcinoma, Merkel cell polyomavirus, UV

## Abstract

Merkel cell carcinoma (MCC) is a rare but aggressive skin cancer with frequent metastasis and death. MCC has a mortality rate of 30%, making it more lethal than malignant melanoma, and incidence of MCC has increased almost fourfold over the past 20 years in the USA. MCC has long been considered to be an immunogenic cancer because it occurs more frequently in immunosuppressed patients from organ transplant and HIV infection than in those with immunocompetent. Chronic UV light exposure and clonal integration of Merkel cell polyomavirus (MCPyV) are two major causative factors of MCC. Approximately 80% of MCC are associated with MCPyV, and T cells specific for MCPyV oncoproteins are present in the blood and tumors of patients. Several studies have shown that a subset of MCCs express PD-1 on tumor-infiltrating lymphocytes and express PD-L1 on tumor cells, which suggests an endogenous tumor-reactive immune response that might be unleashed by anti-PD-1 or anti-PD-L1 drugs.

## Background

Merkel cell carcinoma (MCC) is a rare but highly aggressive neuroendocrine skin cancer, which was described for the first time in 1972 as trabecular carcinoma of the skin (1). Based on the ultra-structural proof of neuroendocrine granules and the expression of CK20 and CD56 (2–4), Merkel cells were considered to be the source of MCC. However, the cells of origin of MCC remain a controversial issue. Recent studies have suggested the origin of MCC may reside in epidermal/dermal stem cells in the dermis (5) or in precursor B cells (6, 7). The incidence of MCC is rising steadily and more than one-third of patients die of MCC, making it twice as lethal as malignant melanoma (8). Risk factors for MCC include fair skin, chronic sun exposure, chronic immune suppression, and advanced age (9–12). In the USA, age-adjusted incidence increased from 0.15 to 0.44 per 100,000 from 1986 to 2004 (13). Consistent with other UV-related skin cancers, incidence rate of MCC in Queensland, Australia is higher than those in the rest of the world (age-adjusted incidence of 1.6 per 100,000) (14). The incidence of MCC in Asia is thought to be low, although no population-based data are available (15, 16). The majority of MCC is associated with Merkel cell polyomavirus (MCPyV), while the remaining is triggered by UV-mediated mutations (17, 18). MCPyV DNA integrates into the host genome of approximately up to 80% of MCCs in the northern hemisphere, whereas its presence is much lower in other geographic regions such as Australia (~30%) (17, 19). Since several lines of evidence indicate the outstanding immunogenicity of MCC, irrespective of MCPyV integration, immune modulating treatment strategies are particularly attractive. Promising results from immune checkpoint inhibitor therapy in first and second line are now available, which expands the treatment armamentarium for MCC patients.

## Clinical and Histological Features

Merkel cell carcinoma presents as a firm, painless, rapidly enlarging, red-violet cutaneous nodule with a smooth surface. The most frequently affected site is the head and neck region (50%), followed by the trunk (30%) and the limbs (10%), although MCC may arise in any body site, including the mucosae (20–22). Heath et al. developed the AEIOU acronym to define the clinical features associated with MCC: asymptomatic/lack of tenderness, expanding rapidly, immune suppression, older than age 50, and UV-exposed site on a person with fair skin. In a study of 195 patients, 89% presented with three or more of the AEIOU characteristics (23). MCC originates in the dermis and only occasionally exhibits an epidermal involvement. Histopathological characteristics of MCC include a monotonous population of tumor cells with large prominent nuclei and scant cytoplasm (24). Immunohistochemically, MCC is positive for EMA, CK20 with a perinuclear dot staining pattern, and neuroendocrine markers including synaptophysin and chromogranin (3, 25–27). Metastatic pulmonary small cell carcinoma can be excluded when the tumor cells prove negative for TTF-1 (28). Unknown primary MCC, which usually presents clinically positive nodal disease with unidentified primary tumor, are likely to have a significantly improved survival compared to those with concurrent primary tumor (29–32). Recent reports showed that unknown primary MCC had higher tumor mutational burden and lower association with MCPyV than those with known primary (33), In addition, nodal tumors from unknown primary MCC contained abundant UV-signature mutations (33), suggesting underlying immunological mechanism between regression of primary tumor and better prognosis of unknown primary MCC.

## Etiology

Like Kaposi’s sarcoma, immunocompromised patients with T-cell dysfunction are more likely to be affected by MCC. For example, patients with AIDS have an incidence rate that is 11–13 times greater compared with the general population (11), and solid organ transplant recipients are 5–10 times more likely to develop MCC (34, 35). Also, case reports have described spontaneous regression of MCC tumors after biopsy or an improvement in immune function, further indicating a link to the immune system (36–39). These data collectively suggested that MCC may be linked to a pathogen and in 2008, MCPyV was discovered, and it is now clear that this virus plays a key role in the majority of MCC cases (17).

Merkel cell polyomavirus is a member of the polyomavirus family comprised of non-enveloped, double-stranded circular DNA viruses. MCPyV-specific antibodies have been detected in 9% of children under 4 years of age, 35% of teenagers, and 80% of individuals 50 years or older (40), suggesting that it may be part of the cutaneous microbiome (41). Interestingly, despite this high prevalence, MCPyV has not been shown to cause any disease other than MCC (42). MCPyV-related oncogenesis requires integration of the viral genome into the host-genome and mutation of the large T (LT) antigen that is required for viral DNA replication (43). Indeed, MCPyV isolated from MCCs, in contrast with MCPyV from non-tumor sources, present mutations that are responsible for the premature truncation of the MCV LT helicase (43, 44). These mutations do not affect the Rb binding domain, but eliminate the capacity of the viral DNA to replicate. In this way, the virus loses its capability to replicate in MCC tumor cells, but continues to express motifs that may potentially lead to uncontrolled proliferation (43, 45). Prognostic significance of tumor viral status is still controversial, but the largest cohort study so far including 282 MCC cases (281 cases with available clinical data) showed that, relative to MCPyV-positive MCC patients, MCPyV-negative MCC patients had significantly increased risk of disease progression (hazard ratio = 1.77, 95% confidence interval = 1.20–2.62) and death from MCC (hazard ratio = 1.85, 95% confidence interval = 1.19–2.89) in a multivariate analysis including age, sex, and immunosuppression (46).

Merkel cell carcinoma development is also linked to exposure to UV radiation, and primary MCC lesions preferentially develop on sun-exposed skin (20, 21). The incidence of MCC was determined to be 100-fold greater in patients who underwent PUVA treatment (47). MCPyV-negative MCC is among the most mutated of all solid tumors, including melanoma (18, 48–50). These mutations are mostly UV-signature mutations, such as p53 and Rb, commonly resulting in loss of functional protein expression (18, 49). The high mutational burden in MCC correlates to frequent amino acid changes and large numbers of UV-induced neoantigens (49). Despite significant genetic differences, both MCPyV-positive and -negative MCC exhibit nuclear accumulation of oncogenic transcription factors such as NFAT, phosphorylated CREB, and phosphorylated STAT3, indicating commonly deregulated pathogenic mechanisms (50).

## Treatment

For patients with locoregional MCC, wide excision and/or complete lymph node dissection and/or adjuvant radiation therapy is usually recommended (51). Sentinel lymph node biopsy should be considered for patients with clinically nodal negative patients, although its impact on overall survival is still unclear (51–53).

Although cytotoxic chemotherapy (carboplatin or cisplatin plus etoposide) has been commonly used to treat patients with advanced MCC, responses are rarely durable and few studies have shown a survival benefit (54–57). Early studies showed that levels of intratumoral CD8+ T cells serve as predictors of MCC-specific survival, with 100% survival reported for patients with the highest level of CD8+ infiltrate compared to 60% survival in those with little or no CD8+ infiltration (58, 59). Then MCPyV oncoprotein-specific cells were found to be present in MCC patient blood and enriched in their tumors (60), whose frequency appears to increase with tumor burden (61). Importantly, signs of dysfunction were evident in MCPyV-specific CD8+ T cells from patients, as they expressed both PD-1 and Tim3, suggesting functional exhaustion (61). MCPyV-negative MCC is also associated with high levels of T-cell infiltrates (18). Although both MCPyV-positive and -negative tumor cells express PD-L1, the expression levels of PD-L1 in virus-positive tumors seem to be higher than those in virus-negative tumors (18, 62). These findings, therefore, provide rationale for immunotherapy targeting the PD-1 pathway in advanced MCC.

A multicenter, phase 2, non-controlled clinical trial studied pembrolizumab (anti-PD-1 Ab) 2 mg/kg every 2 weeks in 26 patients with advanced MCC who had not received prior systemic therapy. The objective response rate (ORR) to pembrolizumab among the 25 patients with at least one evaluation during treatment was 56% including a 16% complete response (CR) rate. Of the 14 responsive patients, the response duration ranged from at least 2.2 months to at least 9.7 months. Overall, the trial had an estimated progression free survival (PFS) of 67% at 6 months. Pembrolizumab was effective in both MCPyV-positive and -negative tumors (ORR 62 and 44%, respectively, not significantly different) (63). The preliminary data from this trial led to pembrolizumab being listed as a treatment option for disseminated disease in the 2017 NCCN guidelines for MCC (64).

A multicenter, international, open-label, phase 2 clinical trial studied avelumab (anti-PD-L1 Ab) in 88 patients with distant metastatic disease who had previously received at least one line of chemotherapy. This trial found an ORR of 33% with a CR rate of 11%. At 6 months, PFS was 40% and the estimated PFS at 1 year was 30%. As with pembrolizumab, avelumab was found to be effective in both MCPyV-positive and -negative tumors (ORR 26 and 35%, respectively, not significantly different) (65). Based on these results, FDA granted an accelerated approval for avelumab as first-line treatment of patients with metastatic MCC in March 2017. In the avelumab trial, a trend toward a higher response rate was observed in patients with fewer lines of prior treatment, which along with the pembrolizumab data strongly suggest that immunotherapy targeting the PD-1 pathway should be considered for first-line treatment in patients with advanced MCC.

An international, single arm, open-label trial of nivolumab (anti-PD-1 Ab) 240 mg/body every 2 weeks included both patients who had and those who had not received prior chemotherapy (36 and 64%, respectively) is ongoing (NCT02488759; CheckMate358). In this study, 15 of 22 patients (68%) had objective responses, and PFS at 3 months was 82%. Responses occurred in 10 of 14 treatment-naive patients including 3 CR, in 5 of 8 patients including 5 partial responses with 1–2 prior systemic therapies (63%) (Table 1). Based on the preliminary data from this trial, nivolumab was listed along with avelumab and pembrolizumab as a treatment option for disseminated disease in the 2018 NCCN guidelines for MCC (51).

**Table 1:** Ongoing clinical trials in MCC (http://ClinicalTrials.gov).

NCT identifier	Title	Phase	Intervention
NCT03071406	Randomized Study of Nivolumab + Ipilimumab ± SBRT for Metastatic Merkel Cell Carcinoma	2	Nivolumab Ipilimumab SBRT
NCT02643303	A Phase 1/2 Study of *In Situ* Vaccination with Tremelimumab and IV Durvalumab Plus PolyICLC in Subjects with Advanced, Measurable, Biopsy-Accessible Cancers	1/2	Durvalumab Tremelimumab Poly ICLC
NCT02488759	An Investigational Immuno-therapy Study to Investigate the Safety and Effectiveness of Nivolumab, and Nivolumab Combination Therapy in Virus-Associated Tumors (CheckMate358)	1/2	Nivolumab Ipilimumab BMS-986016 Daratumumab
NCT02584829	Localized Radiation Therapy or Recombinant Interferon Beta and Avelumab with or without Cellular Adoptive Immunotherapy in Treating Patients with Metastatic Merkel Cell Carcinoma	1/2	Avelumab Merkel cell polyomavirus TAg-specific polyclonal autologous CD8-positive T cells Interferon beta, RT
NCT03271372	Adjuvant Avelumab in Merkel Cell Cancer (ADAM)	3	Avelumab
NCT02196961	Adjuvant Therapy of Completely Resected Merkel Cell Carcinoma with Immune Checkpoint Blocking Antibodies Versus Observation (ADMEC-O)	2	Ipilimumab Nivolumab

## Conclusion

Advanced MCC is generally considered to be sensitive to chemotherapy, but responses are transient, offering a median PFS of only 3 months (55). On the other hand, although no randomized trials compare chemotherapy with immunotherapy, data from treatment with immune checkpoint inhibitors are promising with responses both in MCPyV-positive and -negative MCC, although nearly half of patients do not derive durable benefit from these drugs. Now that avelumab has been approved for treatment of advanced MCC in the USA, EU, and Japan, the spectrum of current therapy for patients with MCC is changing. Several clinical trials of immune checkpoint inhibitors (anti-PD-1, PD-L1, and CTLA-4 Abs) administered as monotherapy or in combination with other agents or modalities are ongoing (Table 1) and may provide further treatment options for patients with advanced MCC in the near future.

## Author Contributions

The author confirms being the sole contributor of this work and approved it for publication.

## Conflict of Interest Statement

The author declares that the research was conducted in the absence of any commercial or financial relationships that could be construed as a potential conflict of interest.
